# Psychological stress and cancer: new evidence of an increasingly strong link

**DOI:** 10.37825/2239-9747.1010

**Published:** 2020-10-01

**Authors:** M Abate, M Citro, M Caputo, S Pisanti, R Martinelli

**Affiliations:** 1 Department of Medicine, Surgery and Dentistry “Scuola Medica Salernitana” University of Salerno, Via S. Allende, 84081, Baronissi, Salerno, Italy

**Keywords:** Cancer prevention, Psychological stress, Inflammation

## Abstract

To date stress, a highly complex process that disrupts homeostasis and involves environmental and psychosocial factors, is considered as one of the most crucial factor that affects our daily life, especially urban dweller’s life.

Clinical and experimental studies widely support the notion that adrenergic stimulation due to chronic stress affects inflammation and metabolism.

In this work, supported by several recent scientific evidences, we show how stress plays a positive role in cancer initiation, progression and cancer metastasis, a negative role for anti-tumor immune function and therapy response.

Understanding the intricacies of this interaction could provide an additional help on how act in cancer prevention and therapy.

## I. INTRODUCTION

In the modern civilization stress is considered as one of the most crucial factor that affects our daily life. Stress was described for the first time by the endocrinologist Hans Seyle in 1936 as the human body’s biological response to adverse changes in the environment such as physical, mental and social stimuli called “stressors”. To address and overcome these challenges and preserve the wellness our body has evolved a complex repertoire of physiologic and behavioral changes leading to adaptive stress response [1]. Based on their duration and intensity, the stress responses are generally grouped into two major types: acute and chronic. Acute stress, generally considered to be healthful for the organisms is a transient condition while prolonged chronic stress may elicit a disruption of internal homeostatic mechanisms. Indeed, the human body is able to circumvent and normalize the effects of acute stress, but when it becomes chronic it can disrupt the optimal body equilibrium. Depending on type and severity of the stressors perceived by the organism, stress responses are characterized by specificity in highly interconnected activation of neuroendocrine system. In detail, the hypothalamic-pituitary-adrenal axis (HPA) and the sympathetic nervous system (SNS) are the primary players in the regulation of the stress-related cascade [2, 3].

Stressors inducing the central and peripheral nervous system responses activate HPA that stimulates the release of corticotropin-releasing hormone (CRH) and arginine vasopressine (AVP) from the paraventricular nucleus of the hypothalamus (PVH) which, in turn, enhance the anterior pituitary gland to secrete adrenocorticotropic hormone (ACTH), encephalin and endorphin (END). ACTH mediates the downstream release of glucocorticoids (GC) from the adrenal cortex and catecholamines, norepinephrine (NE) and epinephrine (EPI), from the adrenal medulla. The sustained release of these two neuropeptides results in the activation of systemic stress-responsive pathways that modulate several physiological and biochemical behaviors, including “The Fight-or-Flight Response” and regulation of thermogenesis and cell survival [2, 3]. Conversely, a distressed psychological and physiological state that occurs in individuals in the form of fear, anxiety, pain and depression can lead to a condition of chronic stress bearing adverse effects on cardiovascular and nervous systems and exacerbation of autoimmune diseases.

Moreover, recent epidemiological studies have shown that chronic psychological stress plays a positive role in cancer initiation, progression and cancer metastasis and a negative role for anti-tumor immune function and therapy response [4]. It is recognized that psychological stressors are associated with a greater cancer incidence, especially for breast cancer that is almost ninefold, and at the same time with a poor survival in patients with ovarian, prostate, lung, head and neck, hepatobiliary and lymphoid cancers [4, 5].

## II. CHRONIC STRESS: A ROLE IN CELL SURVIVAL, TUMOR GROWTH AND METASTASIS FORMATION

Catecholaminergic neurotransmitters of the acute and chronic stress responses bind to a class of seven-transmembrane, G-coupled protein receptors: α-adrenergic receptors (α-ARs) and β-adrenergic receptors (β-ARs).

Recently many scientific works shown that several tumor types including pancreatic, colorectal, oesophagus, stomach, lung, breast, ovarian, prostate and nasopharynx cancers, as well as in melanoma, pituitary tumors, Ewing sarcoma, neuroblastoma, and lymphomas are correlated with a non-physiological β-ARs expression [6].

Indeed, various physiological stress model exposing rodents to experimental stressors like as fear or anxiety have been developed and extensively investigated to discover the relationship between β-adrenergic system and tumorigenic processes [7].

Therefore it was possible to observe that, chronic stress promotes the release of stress hormones in murine tumor models increasing tumor burden and aggressiveness of tumor growth [8]. On this light, the molecular mechanisms underlying the effects of stress on tumorigenesis has recently been reviewed. During chronic stress, the activation of β1- and β2-ARs by elevated levels of NE and E promote throught Gs subunits an increasing of cyclic AMP (cAMP) that induces the activation of Protein Kinase A (PKA). As a consequence, PKA can phosphorylate several proteins including cAMP response element-binding protein (CREB), Activating transcription factors (ATF), (Signal transducer and activator of transcription-3) STAT-3, globin transcription factor-1 (GATA-1) and Src family kinases all known to trigger pro-tumorigenic pathways that sustain cellular proliferation, escape from apoptosis, migration and invasion, epithelial–mesenchymal transition (EMT) and pre-metastatic niche formation in different types of cancer [3–5].

Moreover, a recent work suggests that β-ARs have a crucial role in driving neoplastic transformation and tumorigenicity and that chronic stress can enhance the development of acute lymphoblastic leukemia (ALL) via a β-adrenergic signaling pathway with an indirect regulation of pre-B leukemic cells behaviors [9].

Hara et al. have demonstrated that catecholamines over-release, promoting the accumulation of DNA damage and degradation of tumor suppressor p53 in normal cells, could play an important role in cancer development [10].

Therefore, chronic exposure to NE and EPI can have dramatic effects on DNA damage leading to genomic instability and tumorigenesis [11]. The activation of beta-adrenergic system has been associated with a upregulation of the expression of specific oncogenic signaling pathways including Sirtuin-1(Sirt-1) in cervical cancer, Src in ovarian cancer and Human Epidermal Growth Factor Receptor2 (Her2) in breast cancer [12,13].

Chronic stress could contribute to alter tumor survival and cell death induction, indeed recent *in vitro* and *in vivo* experiments show that NE enhances proliferation of gastric cancer cells and promotes autophagic flux which has a tumor-promoting role through AMP-activated protein kinase-unc-51-like kinase-1 (AMPK-ULK1) pathway [14].

Moreover NE stimulates cell proliferation and other cancerous biological behaviors of pancreatic ductal adenocarcinoma (PDAC) cells via β-adrenergic receptor-dependent activation of p38 mitogen-activated protein kinases (p38/MAPK) pathway [15], and at the same time was able to inhibit apoptosis and promote cell survival of cancer cells, binding β2-ARs in a Notch-1-dependent manner [16].

Furthermore, EPI reduces the sensitivity to apoptotic cell death mechanism in prostate and breast cancer cells through interaction with β-ARs and in turn the β-ARs/cAMP/PKA signaling pathway promotes the phosphorylation and consequent inactivation of pro-apoptotic protein BCL2-associated death promoter (BAD) [17] confirming that signaling through β-adrenergic receptor can enhance tumor survival through multiple intracellular pathways.

In addition to a role in cell survival and tumor growth, chronic stress has also been widely studied for mediating metastasis. A sequence of molecular events are used to delineate the process including cancer cell local invasion, extravasation, micrometastasis formation and colonization and the activation of β-adrenergic system seems to be involved in each step of the cancer invasion-metastasis cascade [18].

Tumor cell adhesion to the components of extracellular matrix (ECM) is a determinant key to cell migration, invasiveness of cancer cells and their metastatic spread from the primary tumor to surrounding tissues. The β-adrenergic system activation takes part in tumor cell adhesion and facilitates migration and invasion.

Recent work shows that the permanent stimulation of β2-ARs modulates the integrin-mediated cell adhesion to fibronectin through a molecular mechanism that involves the exchange protein directly activated by cAMP (Epac) and Rap1, a small GTPase belonged to Ras oncogene proteins [19]. It has indeed been highlighted that, the activation of focal adhesion kinase (FAK) by NE and EPI through a β-ARs/PKA/Src dependent pathway protected ovarian cancer cells from anoikis, a detachment-induced apoptosis from surrounding ECM and neighboring cells [20].

Regarding the trans-endothelial invasion process, several studies suggest that catecholamines may potentiate the ability of tumor cells to interact with vascular endothelium promoting this process and subsequent metastasis. To support this findings an *in vitro* study has demonstrated that NE was able to promote adhesion of breast cancer cells to human pulmonary microvascular endothelial cells and trans-endothelial invasion involving the susteined release of growth-regulated oncogene α (GROα) and β1-integrin pathway [21].

In 2015, Barbieri et al. have shown that NE was able to influence *in vitro* and *in vivo* metastatic potential of prostate cancer cells through β2-ARs cell signaling. In fact in their experiments the treatment with NE conducted in mice xenografted with human hormone-independent prostate cancer cell line significantly increased migration of these cells from the primary tumor into inguinal lymph nodes, consequently to formation of novel metastatic foci. Additionally, it has been observed that NE induced overexpression of cytokeratin (CK), matrix-metalloproteinase-2 (MMP-2) and -9 (MMP-9) that was paralleled by EMT effects and correlated to higher mobility and invasive behaviors of tumor cells [22].

Taken together, these findings highlight the complex mechanisms by which neurotransmitters and chronic stress exert their effect to promote the growth and spread of cancer.

## III. CHRONIC STRESS AND IMMUNE RESPONSE

It is well known that the adrenergic receptors stimulation by catecholamines significantly increase the TNF-α production, but also increase the concentration of cytokines and interleukins such as IL-6, IL1β, IL10, IL4, IFNγ and C-reactive protein (CRP), that accelerate the progression of inflammatory diseases [23]. Moreover, the sustained release of catecholamines, including norepinephrine, promotes the upregulation and activation of cAMP response element-binding protein (CREB), Phospholipase C, Protein Kinase Cα, Signal transducer and activator of transcription 3 (STAT3), the prolactin hormone and the prostaglandin E2 (PGE2) synthesis, all known to promote proliferation, migration, angiogenesis and apoptosis evasion in several cancers [2–5, 23–25].

New scientific evidence has also shown that cancer cells release neurotrophic factors (Nerve Growth Factor, NGF and Brain-derived Neurotrophic Factor, BDNF) in response to catecholamines that, contributing to cancer innervation, improve tumorigenesis enriching the tumor microenvironment of catecholamines freed from the newly formed nerve endings [26].

Furthermore, norepinephrine released from nerve terminals through the adrenergic receptors is able to interact with immune cells. Recently, Ambree et al. (2018) showed that this property correlates with an altered cell-mediated immunity in a mouse model of lymphoma. Indeed, catecholamines and glucocorticoids have been reported to damage the antigen presentation process, affect T cell proliferation, and attenuate humoral and cell-mediated immunity. It has been shown that noradrenaline, although is able to increase dendritic cell migration in CD8+ T cell-mediated response, greatly reduces their overall ability to efficiently present antigens. Furthermore, the adrenergic stimulation of dendritic cells enhances Th2-associated inflammation and Th17 differentiation, suppressing at the same time the antitumor activity of NK cells and stimulating tumor-associated macrophages to produce pro-inflammatory and pro-angiogenic factors such as the vascular endothelial growth factor (VEGF) and metalloproteinases [27].

## IV. CONCLUDING REMARKS

It is well known as a distressed psychological and physiological state that occurs in individuals in the form of fear, anxiety, pain and depression, easily manifestable with the stressful urban dweller’s life, can lead to a condition of chronic stress bearing adverse effects on cardiovascular and nervous systems and exacerbation of autoimmune diseases and like we show in the present work, there are increasing scientific evidence that underline how chronic psychological stress plays a positive role in cancer initiation, progression and cancer metastasis and a negative role for anti-tumor immune function and therapy response [4]. Moreover, if to the stressful urban dweller’s life are added unhealthy behaviours such as eating and drinking alcohol to excess, smoking, abusing drugs or being physically inactive, the cancer development risk is further incremented [28–31].

At the same time, it is clear that isn’t possible to completely eliminate stress from everyday life but above all from the urban dweller’s life, however, much can be done to reduce it with benefits for physical health.

The only valid solution to get out of a condition of chronic stress is to change, as far as possible, one’s lifestyle.

How?

First and foremost, to cope with stress it is important being aware of one’s sources and causes of stress and only in this way is it possible finding a solution. Certainly the organization and scheduling of daily, weekly and monthly commitments can help to identify priorities and reduce the onset of stress situations. Listening to own physical needs, trying to take care of own health are undoubtedly the basis for having more energy to combat stress conditions, combined with the ability to being able to find time every day to do pleasant things like practicing a sport or relaxing activities. Furthermore, since isolation is one of the factors that can greatly amplify the stress condition, learning to ask for help by confronting other people can reduce stress. Recently, Bonaccio et al. demonstrated how diet can also help reduce stress. The researchers evaluated psychological resilience, that is a measure of stress coping ability, with adherence to the Mediterranean Diet (MD) [32]. MD is already considered one of the most worldwide healthy dietary patterns thanks to a combination of foods rich mainly in antioxidants and anti-inflammatory nutrients with protective effects in reducing oxidative and inflammatory processes of cells, avoiding DNA damages and with contrasting effects on onset, progression and regression cancer [33,34]. In addition to these already well-known beneficial effects of MD, Bonaccio et al. showed that a MD dietary pattern is positively associated with higher psychological resilience, compared to Western-type diets [32].

Furthermore, it is clear that the psychological stress may negatively affect cancer therapy. Therefore, a medical approach more focused on preserving patient psychological wellness, could be able to improve cancer prognosis and treatment at the biological level.

In light of these new findings, in the near future it will be of crucial importance conduct information campaigns to disseminate the latest scientific evidence in order to prevent and minimize the number and the impact on health of common daily stressors and at the same time comply with a medical approach more focused on preserving the patient’s psychological well-being in order to make therapies more effective.
